# An exploration of nucleic acid liquid biopsy using a glucose meter†
†Electronic supplementary information (ESI) available: Experimental section, additional results and discussion and additional figures. See DOI: 10.1039/c8sc00627j


**DOI:** 10.1039/c8sc00627j

**Published:** 2018-03-01

**Authors:** Yu Gu, Ting-Ting Zhang, Zhi-Feng Huang, Shan-Wen Hu, Wei Zhao, Jing-Juan Xu, Hong-Yuan Chen

**Affiliations:** a State Key Laboratory of Analytical Chemistry for Life Science , Collaborative Innovation Center of Chemistry for Life Sciences , School of Chemistry and Chemical Engineering , Nanjing University , Nanjing 210023 , China . Email: weizhao@nju.edu.cn ; Email: xujj@nju.edu.cn

## Abstract

The development of non-invasive techniques for the diagnosis of cancer, characterization of mutation and monitoring treatment response could greatly reduce the morbidity and mortality caused by cancer. Nevertheless, the extremely low amount of cell free nucleic acids makes liquid biopsy a very challenging task. Herein, taking advantage of the pocket size, reliable quantitative results and simple operation of the pocket-sized personal glucose meter (PGM), we report an approach of circulating microRNA-21 (miR-21) detection with high precision and low cost. *Via* target-induced release of invertase from the DNA–invertase conjugate, which could convert sucrose into glucose, the detection of miR-21 in serum was linked to PGM readings. Combining the DNAzyme feedback amplification (DFA) program and highly efficient enzymatic turnover, an ultralow detection limit of 7 × 10^–16^ M for miR-21 was achieved using a PGM as the reporter. The high sensitivity and selectivity of the proposed method meets the requirement of quantifying cell free nucleic acids in serum. In addition, this approach fills the shortage of quantitative RT-PCR and next-generation sequencing in quantifying miRNAs with a short length and greatly reduces the cost of detection. We believe that widely used personal diagnosis devices could hold an important place in the booming area of liquid biopsy.

## Abstract 2

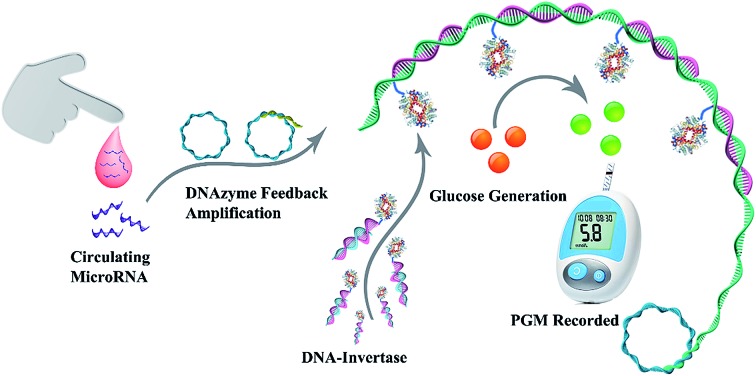
A proof-of-concept strategy for a circulating miRNA assay using a personal glucose meter (PGM) was proposed.

## Introduction

Liquid biopsy, an innovative technology in cancer testing, is a non-invasive approach, which could relieve the suffering of patients from conventional biopsy, decrease the false positive rate because of the tissue sample bias and help oncologists gain a broader molecular understanding of the disease.^1^,^2^ Nucleic acids, including cell free DNA (cfDNA) and circulating mRNA as well as microRNAs (miRNAs), are promising biomarkers in blood for cancer screening, monitoring drug treatment response, and postoperative assessment.^3^–^7^ However, due to the relative rarity of circulating nucleic acids against the huge background in plasma or serum, liquid biopsy still faces challenges including the extraction and enrichment of biomarkers^8^,^9^ and increasing the sensitivity of detection methods.^10^,^11^

Four major technologies for nucleic acid assays are southern and northern blotting,^12^ polymerase chain reaction (PCR),^13^,^14^ DNA microarrays,^15^ and next-generation sequencing (NGS).^16^,^17^ For liquid biopsy targeting trace levels of nucleic acids, quantitative reverse transcription-PCR (qRT-PCR) assay and NGS are the most widely used methods.^18^ qRT-PCR with very high sensitivity requires relatively low amounts of RNA and performs absolute quantification. However, it is more suitable for the quantification of longer DNA/RNA targets instead of miRNAs with a short 20-base length because of the relatively low selectivity during reverse transcription and PCR. The novel NGS systems offer a much larger dynamic range than the PCR, but the sequencing of miRNAs is still limited by the relatively high rate of NGS errors.^19^ In addition, NGS has high requirements for infrastructure, computer capacity and personnel expertise, which limit its clinical application, and the cost for one test is very high ($1000). Therefore, it is meaningful to develop a technology with low requirements for equipment and personnel skills, but high sensitivity and selectivity to nucleic acids for liquid biopsy.

Here we show a proof-of-concept strategy for circulating miRNA assay with a personal glucose meter (PGM). As a widely used personal diagnosis device for point-of-care testing (POCT), a PGM benefits from its pocket size, reliable performance and simple operation. In 2011, Lu's group reported a smart design by combining a PGM with a functional DNA sensor for quantitative detection of targets beyond glucose, including cocaine, adenosine and interferon-gamma.^20^ Inspired by such pioneering work, we started to think about using a PGM in the booming liquid biopsy. The main challenge is the relatively low sensitivity of the PGM for the analysis of circulating nucleic acids. To achieve an ultra-high sensitivity, an amplification strategy of DNAzyme feedback amplification (DFA) was applied, which took advantage of rolling-circle amplification (RCA) and an RNA-cleaving DNAzyme (RCD).^21^,^22^ Unlike the RT-PCR, the whole RCA program could be initiated by a short nucleic acid and generate hundreds of tandemly linked copies of a covalently closed circle in a few minutes under isothermal conditions. The produced RCD elements from RCA triggered the production of more input complexes for RCA. The autonomous reaction circuit resulted in exponential DNA amplification. As suggested by Brennan and Li, sensitivity improvements of 3–6 orders of magnitude over conventional methods could be achieved for miRNA sensing by DFA.^23^ Furthermore, the DFA product was hybridized with DNA-invertase conjugates, and efficiently catalyzed for the hydrolysis of sucrose into glucose, which is quantified by using a PGM. The proposed method provided an ultralow detection limit of 7 × 10^–16^ M for miRNA-21 and a large dynamic range of over 4 orders. Added miRNA-21 in blood serum was determined using the proposed method with high selectivity. The PGM coupled with the DFA program holds promising potential for ultra-sensitive and low-cost liquid biopsy.

## Results and discussion

### Principle

**Scheme 1:**
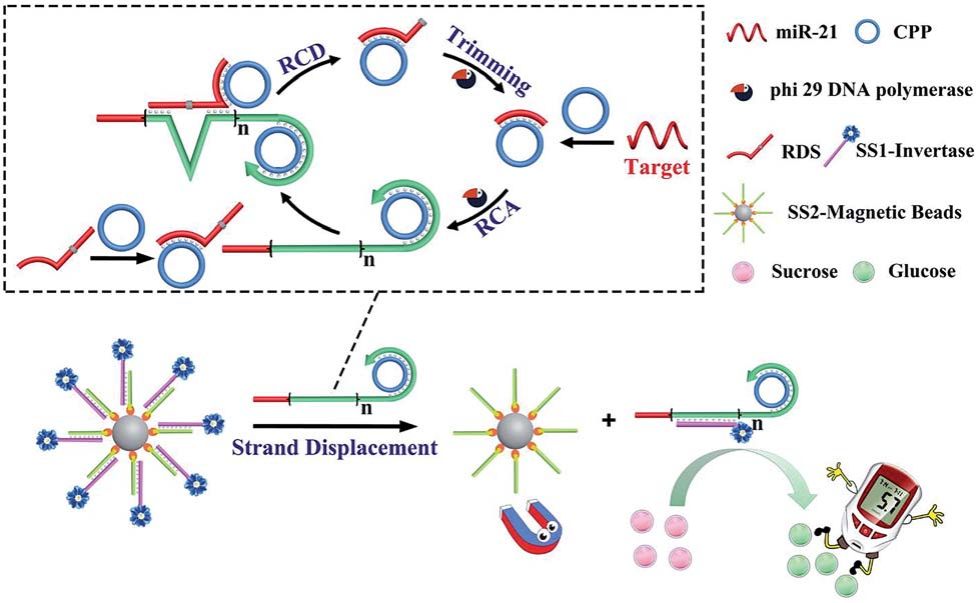
Principle of miR-21 sensing based on DFA and the target-induced release of invertase.

Scheme 1 depicts the working principle of the assay, including the amplification process of DFA, and target-induced release of invertase that converts sucrose into glucose and results in a PGM response. The DFA system consists of a target miRNA, circular padlock probe (CPP) and RNA-containing DNA sequence (RDS) which serves as the substrate of the RCD. These reagents enable the following cascade reactions. Firstly, the target came into contact with the CPP and formed a target/CPP hybrid, which triggered RCA processes in the presence of DNA polymerase (phi29). Secondly, the RDS/CPP complex was hybridized with the RCA product (RP), which resulted in the formation of the RCD. Thirdly, cleavage of the RNA unit of the RDS by the RCD happens which produces the hybrid of the CPP with the cleavage fragment of the RDS. Finally, trimming by phi29, more target/CPP hybrids were produced and fed back into the RCA process. The autonomous DFA system magnifies a single RCA circle for several orders. Furthermore, the DFA product of long single strands containing repetitive complementary CPP units was added to a solution containing DNA–invertase immobilized magnetic beads (MBs). Toehold-mediated strand displacement allowed the release of invertase from the surface of MBs to the solution. After magnetic separation of MBs, the supernatant containing the released invertase was dropped into sucrose solution which converted sucrose into glucose. Ultimately, the concentration of the target was transformed to the PGM reading. Taking advantages of DFA and the target-induced release of invertase from a functional DNA–invertase conjugate, a novel nucleic acid assay with superior performance and a convenient detection technique is expected.

### Validation of the DFA program

**Fig. 1:**
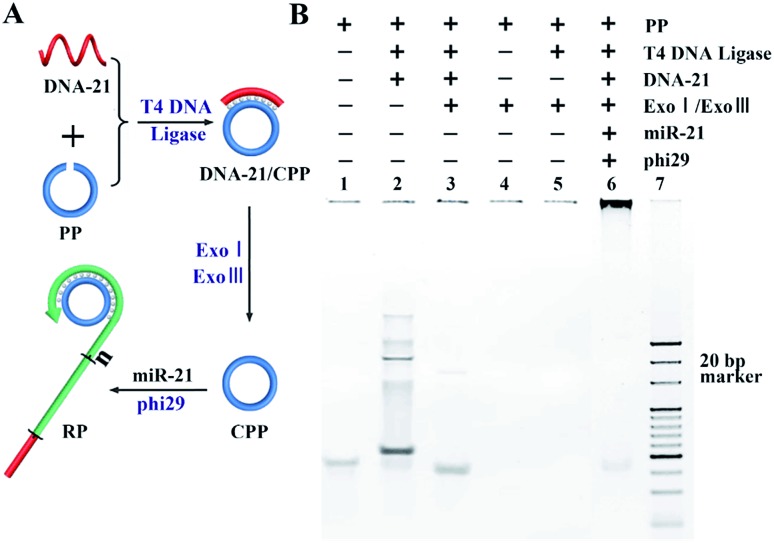
Characterization of the formation of the CPP and RP. (A) An illustration of the synthesis process of the CPP and RCA program. (B) PAGE analysis results for the reactions shown in (A).

For the DFA program triggered by the target miRNA, the CPP and RDS should be designed with two features:^23^ (1) the 5′ portion of the RDS sequence is identical to that of the target miRNA and (2) the CPP contains the antisense sequence of the RDS, as well as the target miRNA. The synthesis of the CPP and RCA was carried out using polyacrylamide gel electrophoresis (PAGE). As shown in Fig. 1, the padlock probe (PP) (lane 1) could be circularized after hybridization with the ligation template DNA-21 and specifically ligated in the presence of T4 DNA ligase, resulting in the main product of the DNA-21/CPP hybrid (lane 2) and some side-products with a higher molecular weight. After adding exonuclease Exo I and Exo III, all side products and dsDNA were digested to deoxy-ribonucleoside monophosphates (dNMPs), with only the CPP (lane 3) remaining. In order to confirm the formation of the CPP, Exo I and Exo III were added to the PP (lane 4) and PP with T4 DNA ligase (lane 5) solution. The results show that without the formation of a circular structure, all added reagents were digested. Finally, miR-21 was added to the synthesized CPP, and RCA was carried out using phi29, producing a long RCA product (RP) (lane 6).

**Fig. 2:**
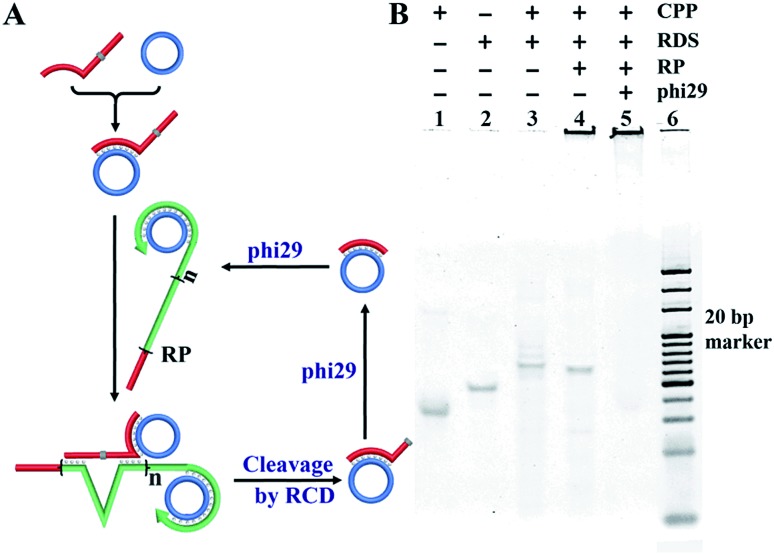
Examination of the RCD process in the presence of the RDS/CPP hybrid and RP. (A) An illustration of the sequential reactions of the cleavage of the RDS within the RDS/CPP hybrid by the RP. (B) PAGE analysis results for the reactions shown in (A).

Furthermore, the formation of the RCD and cleavage events that feed back into the RCA process were examined. As shown in Fig. 2, the CPP (lane 1) was firstly hybridized with the RDS (lane 2) containing its antisense sequence which formed the CPP/RDS hybrid (lane 3). After addition of the RP, the RCD was formed. And the cleavage event occurred, which produced the hybrid of the CPP with the cleavage fragment of the RDS (lane 4). In the presence of phi29, unpaired nucleotides were trimmed, which resulted in the formation of more target/CPP hybrids and fed back into the RP (lane 5). The whole DFA program products were validated by PAGE analysis after 1.5 hours and 3.0 hours (Fig. S1†). With a longer duration, the amount of the long single stranded RP greatly increased, indicating the successful feedback amplification on the basis of RCA.

**Fig. 3:**
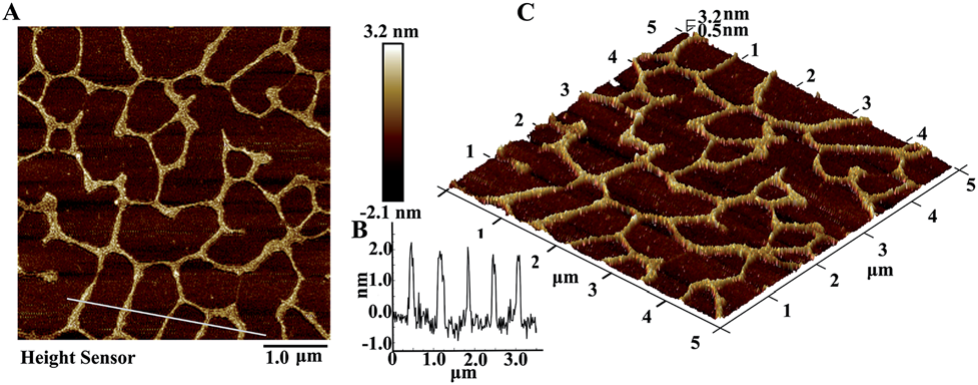
(A) AFM image of the DFA product in tapping mode, (B) the height of the DFA product, and (C) three-dimensional AFM image.

The DFA program products were further characterized by atomic force microscopy (AFM). The DFA products (2.5 h reaction time) after desalination were deposited on an atomically flat mica surface, and monitored in the tapping mode of AFM. As shown in Fig. 3A, the long single stranded DNA exhibited both linear and branched structures which was probably due to its flexibility. And the length of the DFA product could reach up to a few micrometers. The average apparent height of the sheets was measured to be around 1.99 nm (Fig. 3B), suggesting a monolayer of DNA.^24^,^25^ The three-dimensional image of the DNA molecule is shown in Fig. 3C. The height of the long DFA product is uniform.

### Target-induced release of invertase examined using a PGM

For quantitative detection of targets using a PGM, invertase is a perfect mediator, since it could convert sucrose into glucose and sucrose is completely inert in a PGM.^26^ In addition, its highly efficient enzymatic turnover facilitates the sensitivity of detection.^27^ Inspired by Lu's work,^20^ we designed a toehold mediated strand displacement, which could induce the release of the DNA–invertase conjugate from DNA sequences immobilized on MBs, and establish the relationship between the miR-21 concentration and the invertase concentration. Single stranded DNA1 (SS1) and an invertase conjugate (SS1–Inv) was first synthesized. To maintain the catalytic activity of invertase, amine was chosen as the binding site since it was not involved in the active sites of aspartate and glutamate.^30^

**Fig. 4:**
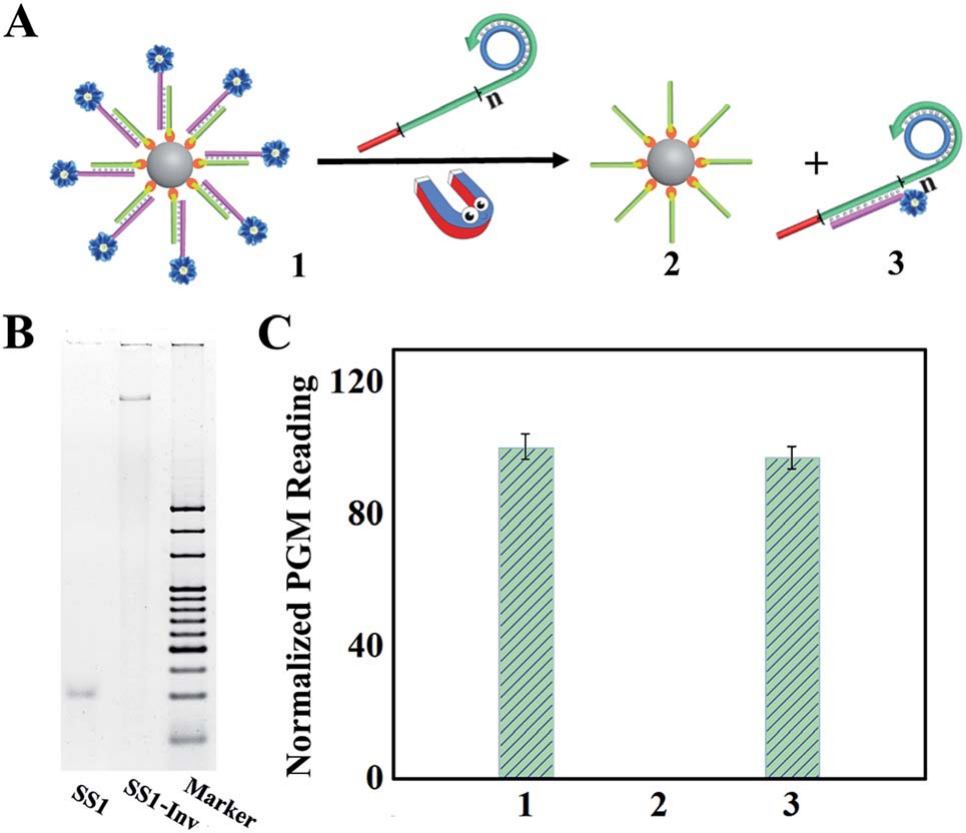
(A) An illustration of the strand displacement process. (B) PAGE analysis of SS1 and SS1–Inv. (C) PGM analysis of the process in (A).

PAGE analysis (Fig. 4B) shows that after the formation of SS1–Inv, a band with a much higher molecular weight was observed compared with that of SS1, indicating that the conjugate was obtained successfully. A PGM was then applied to validate the release of SS1–Inv from MBs. As illustrated in Fig. 4, an excessive amount of DFA product was added to the solution of DNA–invertase–MBs. After strand displacement and magnetic separation, the supernatant maintained 97% enzymatic activity compared to DNA–invertase–MBs, which was examined using the PGM. In contrast, the precipitate rarely contained invertase (no PGM reading), suggesting the successful strand displacement process, which caused the full release of invertase from the DNA sequences on MBs. The strand displacement process was further confirmed by FRET as shown in Fig. S2.†

### Ultrasensitive detection of miR-21 in serum

**Fig. 5:**
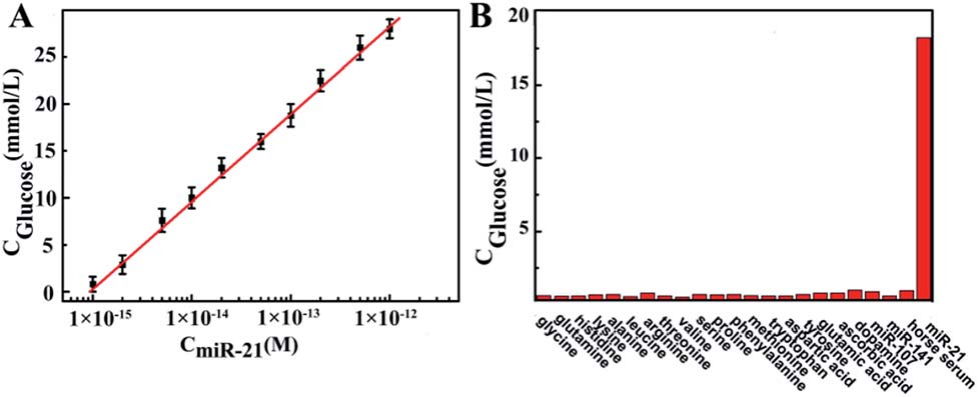
(A) Calibration curve of PGM reading *vs.* miR-21 concentration. (B) PGM readings in accordance with various potential interfering substances. The added concentrations of 17 amino acids correspond to their amounts in serum, which are (from left to right) 300, 400, 100, 200, 300, 160, 100, 150, 150, 150, 300, 100, 16.0, 60.0, 80.0, 100, and 100 μM; the concentrations of ascorbic acid, dopamine, miR-107, and miR-141 are 100 μM, 3.0 nM, 1.0 nM, and 1.0 nM, respectively; horse serum is diluted 10 times; and the concentration of miR-21 is 0.20 pM.

MiRNA expression profiles in serum are related to tumor classification, diagnosis and disease progression.^1^,^7^,^28^ Herein, we demonstrated the PGM determination of circulating miR-21, which was shown to be useful in the detection of breast cancer, prostate cancer and non-small lung cancer.^29^,^30^ Factors which can affect the performance and results of the miR-21 sensing system were optimized, including time of the DFA program, sucrose incubation temperature and time (Fig. S3–S5†). Under optimal conditions, miR-21 was detected using the proposed method. As shown in Fig. 5A, a good linear relationship between the PGM reading and the miR-21 concentration ranging from 1.0 fM to 1.0 pM was achieved. The limit of detection was as low as 0.7 fM according to the PGM detection limit (0.6 mM glucose). It should be noted that the dynamic detection range was restricted to the upper limit of the PGM. Therefore, if we decrease the amount of sucrose added to the product containing invertase, a higher concentration of miR-21 could be quantified. However, since the concentration of circulating miRNA in peripheral blood is mostly lower than 1.0 pM,^31^ the detection range of this method under current conditions is suitable for liquid biopsy. For real analysis of miR-21 in serum or plasma, the specificity of the assay is a key parameter. We tested the selectivity of the PGM coupled to the DFA program toward the potential interfering substances coexisting in serum, including 17 varieties of amino acids, ascorbic acid, dopamine, other microRNAs and 5 times diluted horse serum. As shown in Fig. 5B, these interferences produced negligible PGM readings, suggesting the high selectivity of the proposed method and its potential for application in trace bioanalysis of clinical samples.

To validate the applicability of the proposed method, we tried to do the real sample analysis using human serum. In order to avoid the influence of high viscosity of serum on DFA, we diluted the serum sample 50 times for the recovery test. It should be noted that with dilute serum, the influence from glucose could be ignored. Since the blood glucose level of patients with diabetes usually ranges from 5 mM to 30 mM, after 50 times dilution, even 30 mM glucose can hardly be detected using a PGM (LOD: 0.6 mM). Therefore, the background of glucose doesn't have to be deducted. As shown in Table 1, the recovery of miR-21 (Table 1) ranged from 96.4 to 104.0%, suggesting that this method is a practical tool for the determination of miR-21 in diluted serum samples, with a detection limit of 35 fM.

**Table 1:** Recovery results for the assay of miR-21

Added (×10^–15^ M)	Determined (×10^–15^ M)	Recovery (%)	RSD% (*n* = 3)
100.00	101.8	102.0	4.2
50.00	48.2	96.4	4.0
5.00	5.2	104.0	5.7

## Conclusions

In summary, this study has demonstrated a proof-of-concept strategy for a circulating miRNA assay with a portable PGM. A link between the glucose concentration and the miR-21 concentration was established based on the DNA feedback amplification cascade and DNA-invertase conjugate. A trace amount of miR-21 in serum was greatly amplified *via* the DFA program, resulting in a long stranded DNA product, which induced the release of invertase from the DNA–invertase conjugate, and quantified using a PGM *via* an enzymatic reaction. This method provided an ultralow detection limit of 7 × 10^–16^ M for miR-21 and high specificity for real sample analysis. This approach is universal for nucleic acid liquid biopsy. As long as the DFA program is designed for a specific target, the proposed method could be applied for the assays of other cell free nucleic acids, including circulating tumor DNA, mRNA and other microRNAs. We hope that the application of widely used personal diagnosis devices in liquid biopsy will reduce the worldwide morbidity and mortality caused by cancer.

## Experimental

### Chemicals and materials

Grade VII Inv from baker's yeast (*S. cerevisiae*), sulfosuccinimidyl-4-(*N*-maleimidomethyl)cyclohexane-1-carboxylate (sulfo-SMCC), and tris(2-carboxyethyl)phosphine hydrochloride (TCEP) were purchased from Sigma-Aldrich Co. Ltd. An Amicon Ultra-2 mL 10K was purchased from Merck Millipore Ltd. Streptavidin-coated magnetic beads (MBs, 1.0 μm average diameter) were purchased from Thermo Fisher scientific. All enzymes including T4 DNA ligase, exonuclease I, exonuclease III, deoxynucleoside triphosphates (dNTPs) and phi29 DNA polymerase were obtained from New England Biolabs. Ultrapure water with a resistivity of 18.2 MΩ cm was produced using a Milli-Q apparatus (Millipore) and used in the preparation of all the solutions. Precast-GL gel Native-PAGE (4–15%, 10 well), the UNIQ-10 Column Micro-DNA Gel Extraction Kit, and all the DNA and microRNA molecules reported in this work are synthesized by Sangon Biotech. (Shanghai, China). All other reagents were of analytical grade. The sequences of the DNA and microRNA molecules are described (Table 2):

**Table 2:** Oligonucleotide sequences used in this work

Name	Sequences (5′–3′)
miR-21	UAGCUUAUCAGACUGAUGUUGA
miR-141	UAACACUGUCUGGUAAAGAUGG
miR-107	ACUAUCGGGACAUGUUACGACGA
DNA-21	TAGCTTATCAGACTGATGTTGA
PP	TCT GAT AAG CTA CCT AGC ATA GCC TCC CAA AAT ATC CTA TAT TTC GGC CCC GACCTG GTT CGA TAT CTC A AC ATC AG
RDS	TAG CTT ATC AGA CTG ATG TTG ATT TTT TTT TTT TAC TCT TCC TAG CTrA TGG TTC GAT CAA GA/3InvdT
MRP	CTG ATG TTG AGA TAT CGA ACC AGG TCG GGG CCG AAA TAT AGG ATA TTT TGG GAG GCT ATG CTA GGT AGC TTA TCA GA
FAM–SS1	AAGCTACCTAGCATAGCCTCCCAAAATATCCTATA/i6FAMdT/TTCGGCCCCGAC
Dabcyl–SS2	AAAAAAAAGCA/iDabcyldT/ATAGGATATTTTGGGAGGCTATGCTAAT
SH–SS1	SH/AAGCTACC TAG CAT AGC CTC CCA AAA TAT CCT ATA T TTCGGCCCCGAC
Bio–SS2	Bio/AAAAAAAAGC ATA TAG GAT ATT TTG GGA GGC TAT GCTA AT

### Apparatus

The fluorescence emission spectra were obtained on a Shimadzu fluorescence S-3 spectrophotometer (RF-5301PC, Shimadzu Co., Japan). Atomic force microscopy (AFM) images were recorded on a 5500 Atomic Force Microscope (Agilent Technologies, USA). The glucose concentration was recorded on a Roche ACCU-CHEK performa Nano personal glucose meter, and the PGM readings were corrected using the standard glucose solution (Fig. S6†).

#### Synthesis of the SS1–Inv conjugate

Invertase and SS1 were combined using sulfo-SMCC, a water-soluble, double functional group crosslinking reagent that possessed a negatively-charged sulfonate group on the *N*-hydroxysuccinimide ring. It could link invertase with the amide bond, and couple to SH–SS1 by the maleimide end at pH 7.4. This conjugate was prepared and purified according to a reported procedure with slight modifications.^20^ Briefly, Inv (2.0 mg) and sulfo-SMCC (1.0 mg) were dissolved in PBS buffer (1 mL, 10 mM sodium phosphate, 145 mM NaCl, 2.7 mM KCl, pH = 7.4) and shook for 2.5 h (1000 rpm). The product of Inv–SMCC was re-dissolved in 500 μL PBS (10 mM, 145 mM NaCl, 2.7 mM KCl, pH 7.4) after being purified using an Amicon-10K. Meanwhile, TCEP actived SH–SS1 (120 μL, 0.1 mM) was mixed with the Inv–SMCC and vigorously stirred overnight at 30 °C. The resultant Inv–SS1 was purified using an Amicon-10K and kept at a 5 mg mL^–1^ calculated concentration at 4 °C before use.

#### Preparation of the SS1–Inv/SS2–MB complex

Streptavidin coated MBs (40 μL 10 mg mL^–1^) were pre-washed 6 times and resuspended in 100 μL TKMg buffer (20 mM Tris–HCl, 10 mM (NH_4_)_2_SO_4_, 50 mM KCl, 2 mM MgSO_4_, 0.1% Tween 20, pH 8.8) before being mixed with biotin–SS2 (20 μL of 75 μM) for 1 h on a vertical rotator. Then the unreacted substance was washed 6 times and redispersed in 50 μL TKMg buffer. Finally, 10 μL Inv–SS1 was incubated with SS2–MBs for 2 h while stirring, followed by washing 6 times and storing at 4 °C at a concentration of 0.2 mg mL^–1^ (calculated using invertase).

#### DFA reactions

The DNA strands and miRNAs were dissolved in 10 mM Tris–HCl buffer and diethyl pyrocarbonate (DEPC) treated water with a concentration of 100 μM at –20 °C, respectively. Before DFA reactions, all the DNAs were heated at 95 °C for 5 min and then gradually cooled down to room temperature. The DFA process is described briefly as follows: 10 μL of PB (10 μM) was first mixed with 10 μL DNA-21 (10 μM), heated to 95 °C for 5 min, cooled at room temperature for 30 min. To this mixture were added 5 μL of 10× T4 DNA ligase buffer, 1 μL 400 K units per mL of T4 DNA ligase, and water to a total volume of 50 μL. The mixture was incubated at 16 °C overnight before being heated at 65 °C for 10 min. Then, to the reaction solution were added 1 μL 20 K units per mL exonuclease I and 1 μL 100 K units per mL exonuclease III and the mixture was incubated for 2 hours at room temperature before being heated at 80 °C for 20 min, and thus the circular padlock probe (CPP) was synthesized. Before the DFA reaction, 10 μL RCD (2 μM) was first mixed with 10 μL CPB for 2 h at 37 °C to form a complex of RCD–CPB. The DFA experiments were carried out in the 50 μL 1× phi29 DNA polymerase reaction buffer containing 10 μL RCD–CPB complex, 1 μL CPB, 1 μL dNTPs (10 mM), 1 μL BSA (10 mg mL^–1^), 1 μL phi29 DNA polymerase (10K units per mL) and 10.0 μL of target miR-21 (varying concentrations), followed by incubation at 30 °C for 2.5 h before being heated to 65 °C for 10 min.

#### Detection of miR-21 with the PGM

100 μL Inv–SS1/SS2–MBs was placed in a magnetic rack for 2 min, and the clear solution was discarded, followed by adding 50 μL of DFA solution produced with varying concentrations of miR-21 and incubating at 37 °C for 2 h, then the solution was placed close to the magnetic rack. The clear supernatant was transferred to 50 μL of sucrose (1 M) and heated to 55 °C for 60 min before PGM measurements.

#### Gel electrophoresis analysis

In the denaturing gradient gel electrophoresis assay, the reaction products were analyzed using a Bio-Rad ChemiDoc MP Imaging System. 10 μL of the sample and 2 μL of 6× loading buffer were applied to individual lanes in 4–15% polyacrylamide gel. Electrophoresis was run at 100 V for 5 min and 150 V for 40 min in 1× TBE buffer (9 mM Tris–HCl, 9 mM boric acid, 0.2 mM EDTA, pH 7.9) at room temperature. Then the gel was placed in 100 mL 1× TAE, containing 10 μL 10 000× gel green for 1 min and photoed using a gel image analysis system.

## Conflicts of interest

There are no conflicts to declare.

## Supplementary Material

Supplementary information
