# Supplementary material for: An exploration of nucleic acid liquid biopsy using a glucose meter
Source: Chem Sci. 2018 Mar 1;9(14):3517–22. doi: 10.1039/c8sc00627j (PMC5934745; doi:10.1039/c8sc00627j)
Supplement: Supplementary file 1 [file SC-009-C8SC00627J-s001.pdf]

Electronic Supplementary Material (ESI) for Chemical Science.
This journal is © The Royal Society of Chemistry 2018

Supporting information for

An Exploration of Nucleic Acid Liquid Biopsy Using Glucose Meter

Yu Gu, Ting-Ting Zhang, Zhi-Feng Huang, Shan-Wen Hu, Wei Zhao*, Jing-Juan Xu* and Hong-Yuan Chen

State Key Laboratory of Analytical Chemistry for Life Science and Collaborative Innovation Center of Chemistry for Life Sciences, School of Chemistry and Chemical, Engineering, Nanjing University, Nanjing 210023, China.

*Corresponding Author: Phone/fax: +86-25-89687294. E-mail: weizhao@nju.edu.cn (W. Zhao); xujj@nju.edu.cn (J.J. Xu).

Table of contents:

1. **PAGE analysis of DFA** 3

2. **Validation the toehold mediated strand displacement.** 4

3. **Optimization of the incubation temperature and time for enzymatic catalysis.** 5

4. **Optimization of the time course of DFA cascade.** 6

5. **Calibration of the glucose meter** 7

1. PAGE analysis of DFA

PAGE analysis was applied to characterize the whole process of DFA. As shown in Figure S1, after phi29 was added in miR-21/CPP hybrid (lane 1), RCA was carried out and produced long RCA product (RP) (lane 1). When RDS was added in the system, the feedback amplification process was triggered. Within 0.5 h, the band of RP showed no obvious change (lane 3). However, along with the increase of DFA reaction time to 1.5 h, the products containing large molecular-weight DNA significantly increased (lane 4, lane 5), indicating the successful feedback amplification on the basis of RCA. As incubation time was greater than 1.5 h, the intensity response barely changed.

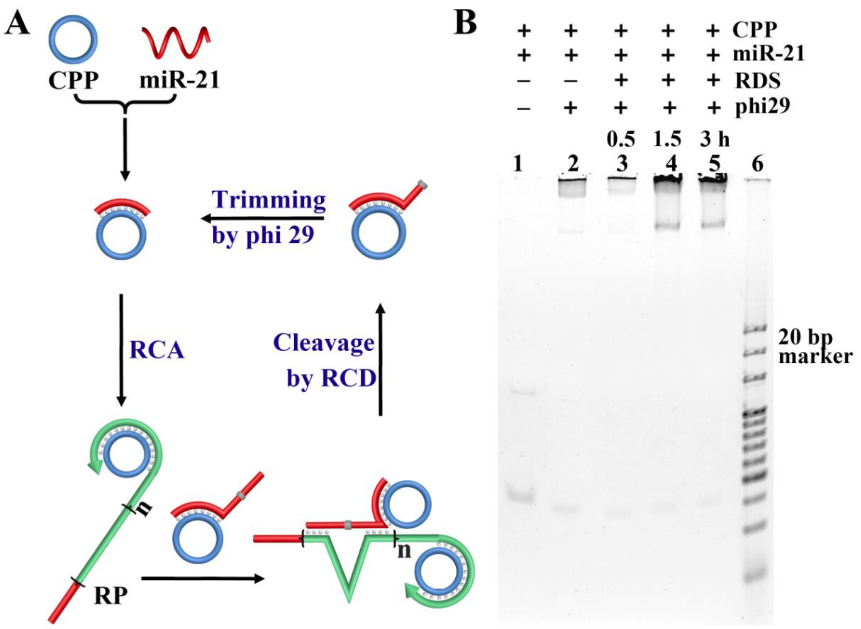

Figure S1. (A) Scheme of DFA program, (B) PAGE analysis for the cascade reactions shown in (A).

2. Validation the toehold mediated strand displacement.

In order to validate the toehold mediated strand displacement system of DFA products with SS1-Invertase/SS2-MBs conjugates, a fluorescence resonance energy transfer (FRET) experiment was designed based on MRP (monomer RCA product). As illustrated in Figure S2A, the fluorescence of FAM-SS1 was initially quenched by Dabcyl-SS2. Once MRP triggered the strand displacement process, the strand labeled with dabcyl quenching group was released into solution, resulting in fluorescence recovery. Fluorescence spectrum of the above DNA strands was shown in Figure S2B, expected fluorescence recovery of FAM-SS1 was determined, indicating the occurrence of strand displacement.

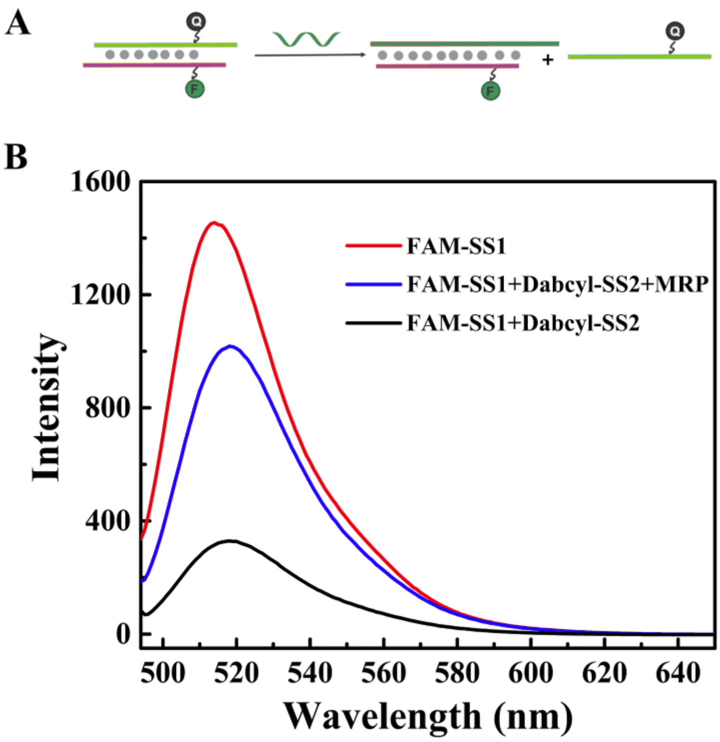

Figure S2. (A) Schematic illustration of fluorescence recovery triggered by strand displacement, (B) Fluorescent spectra of FAM-labeled SS1, FAM-SS1/Dabcyl-SS2 hybrid before and after addition of MRP.

3. Optimization of the incubation temperature and time for enzymatic catalysis.

The conditions of enzymatic catalysis are important factors which can affect the performance of PGM sensing for other targets. Firstly, invertase was mixed with sucrose at different temperature and the glucose produced was detected by PGM. As shown in Figure S3, the enzymatic reaction was found to be applicable in the temperature range of 40~65 ^{o}C, and 55 ^{o}C was chosen in the subsequent experiments as the optimal temperature.

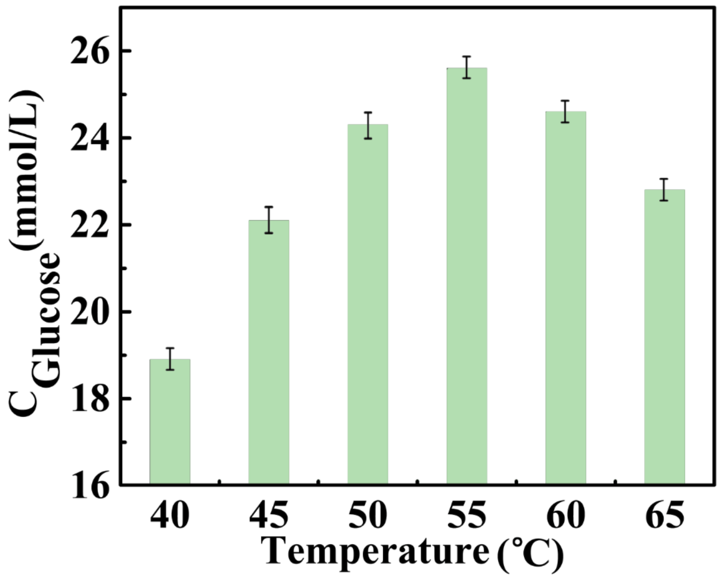

Figure S3. PGM reading to sucrose incubated with invertase at different temperature.

Further, the reaction time of enzymatic catalysis was optimized using three concentrations of invertase and 1.0 M sucrose. As shown in Figure S4, with high concentration of invertase of 0.25 mg/ml and 0.5 mg/ml, the PGM reading quickly increased within the initial 35 min, at then kept stable. With lower concentration of 0.1 mg/ml, the product reached stable after 60 min. We suppose when the concentration of invertase was lower than 0.1 mg/ml, the stable time could be longer. However, considering the efficiency of detection, 60 min was selected as the optimized time.

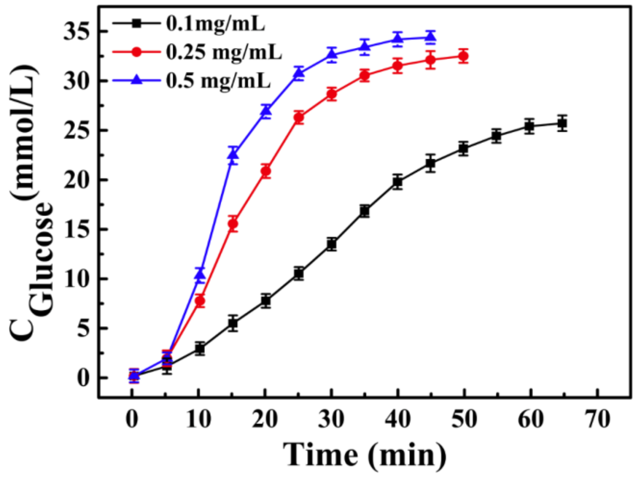

Figure S4. Time dependence of enzymatic reaction with different concentrations of invertase

4. Optimization of the time course of DFA cascade.

Although PAGE analysis confirmed long reaction time of DFA resulting in more long RCA products, it couldn't be applied for quantification. Here we used PGM as the readout for DFA time optimization. As shown in Figure S5, the PGM reading increased rapidly at the first 150 min, and then became consistent. Therefore, 150 min incubation time for DFA system was chosen in subsequent experiments.

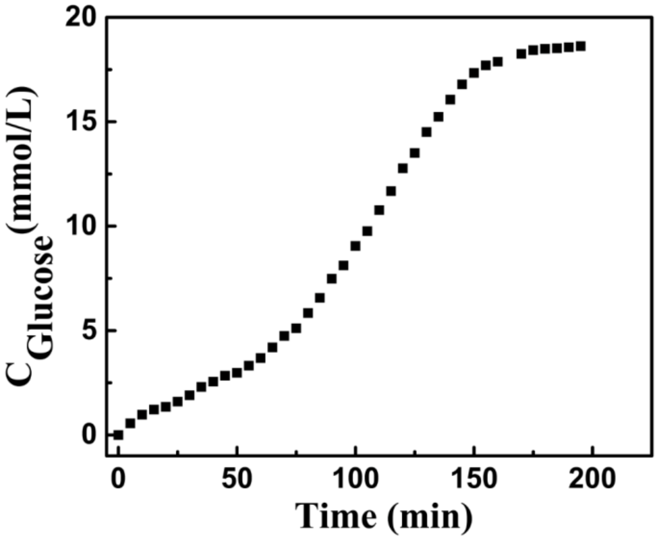

Figure S5. Time dependence of DFA cascade using PGM readout.

5. Calibration of the glucose meter

PGM has been calibrated by standard glucose solution, which shows negligible error of glucose concentration.

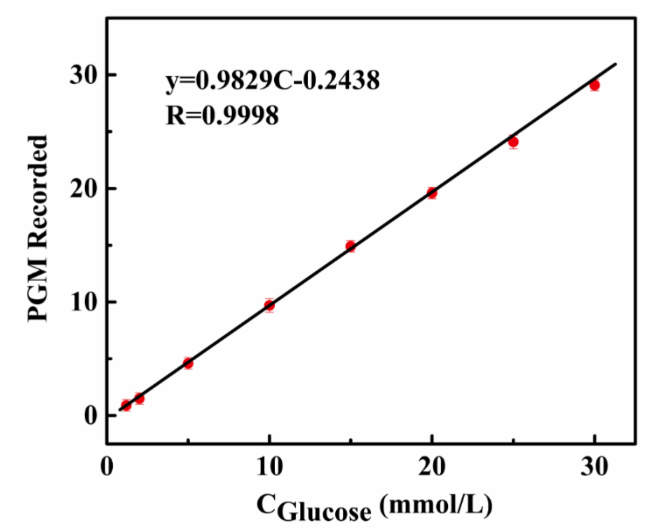

Figure S6. PGM recorded vs standard glucose solution.
